# Why Do Situational Interviews Predict Performance? Is it Saying How You Would Behave or Knowing How You Should Behave?

**DOI:** 10.1007/s10869-015-9410-0

**Published:** 2015-06-09

**Authors:** Janneke K. Oostrom, Klaus G. Melchers, Pia V. Ingold, Martin Kleinmann

**Affiliations:** Department of Social and Organizational Psychology, VU University Amsterdam, Van der Boechorststraat 1, 1081 BT Amsterdam, The Netherlands; Institute of Psychology, Erasmus University, Rotterdam, The Netherlands; Institut für Psychologie und Pädagogik, Universität Ulm, Ulm, Germany; Psychologisches Institut, Universität Zürich, Zürich, Switzerland

**Keywords:** Situational interviews, Validity, Behavioral intentions, Ability to identify criteria, Performance

## Abstract

**Purpose:**

The present study examined two theoretical explanations for why situational interviews predict work-related performance, namely (a) that they are measures of interviewees’ behavioral intentions or (b) that they are measures of interviewees’ ability to correctly decipher situational demands.

**Design/Methodology/Approach:**

We tested these explanations with 101 students, who participated in a 2-day selection simulation.

**Findings:**

In line with the first explanation, there was considerable similarity between what participants said they would do and their actual behavior in corresponding work-related situations. However, the underlying postulated mechanism was not supported by the data. In line with the second explanation, participants’ ability to correctly decipher situational demands was related to performance in both the interview and work-related situations. Furthermore, the relationship between the interview and performance in the work-related situations was partially explained by this ability to decipher situational demands.

**Implications:**

Assessing interviewees’ ability to identify criteria might be of additional value for making selection decisions, particularly for jobs where it is essential to assess situational demands.

**Originality/Value:**

The present study made an effort to open the ‘black box’ of situational interview validity by examining two explanations for their validity. The results provided only moderate support for the first explanation. However, the second explanation was fully supported by these results.

## Introduction

The employment interview continues to be the most frequently used predictor in personnel selection practice (Dipboye et al. 2012). Innumerable studies have shown that interviews can be valid predictors of job performance (see Macan 2009; Levashina et al. 2014; Posthuma et al. 2002, for reviews), that they are well accepted by applicants as well as by recruiters (e.g., Lievens et al. 2005), and that they show less subgroup differences than other frequently used selection instruments (e.g., Huffcutt et al. 2001).

A commonly employed structured interview format that has received considerable attention in the literature is the situational interview (Latham et al. 1980). Situational interviews present applicants with hypothetical situations that are derived from systematic analyses of job requirements. Specifically, they present applicants with work-related dilemmas in which the desired reactions are not easily discerned and ask applicants what they would do if they were actually confronted with these situations (Latham and Saari 1984). Situational interviews have been found to be one of the most criterion-valid interview techniques. Accordingly, several meta-analyses found mean corrected validities between 0.41 and 0.47 for situational interviews (Huffcutt et al. 2004; Latham and Sue-Chan 1999; Taylor and Small 2002).

However, it is still unclear why situational interviews predict performance. The most obvious explanation for situational interview validity is that the ratings of the dimensions that they are designed to measure are relevant for the future job (Huffcutt 2011). However, research testing the internal construct-related validity of interviews provides inconclusive evidence for whether situational interviews measure the dimensions or constructs they are intended to measure (e.g., Conway and Peneno 1999; Huffcutt et al. 1996, 2001; Melchers et al. 2009).

Since assessing the intended job-relevant constructs does not appear to account for the validity of situational interviews, several researchers have called for empirical evidence regarding the underlying mechanisms of situational interviews and interviews in general (e.g., Macan 2009; Huffcutt 2011; Maurer et al. 1999; Ryan and Ployhart 2014). Identifying these underlying mechanisms will not only help understanding situational interview validity, but might contribute to their advancements and it would also help deciding on which other predictors to use in the assessment of potential job candidates (Klehe and Latham 2006).

Therefore, the goal of the present study was to shed light on why situational interviews predict work-related performance by examining two explanations for their criterion-related validity that have been proposed in the literature. The first of these explanations assumes that situational interviews measure interviewees’ behavioral intentions (e.g., Latham 1989; Latham et al. 1980). The second explanation assumes that situational interviews measure interviewees’ ability to identify criteria (ATIC), that is, whether interviewees are able to correctly decipher the situational demands they are faced with in social situations (cf. Kleinmann et al. 2011). Below, we describe the explanations for situational interview validity in more detail.

## Behavioral Intentions and the Validity of Situational Interviews

A main explanation that has long been offered for the validity of situational interviews is that they assess behavioral intentions (e.g., Latham 1989; Latham et al. 1980). Intentions, a core variable in social cognitive theory (Bandura 1986), are assumed to capture the motivational factors that influence behavior and to indicate how hard people are willing to try or how much effort they would exert to perform certain behaviors (Ajzen 1991). Hence, intentions are viewed as the direct motivational instigator of behavior (Fishbein and Ajzen 1975; Locke and Latham 1990). Accordingly, a meta-analysis by Armitage and Conner (2001) showed a substantial correlation (*r* = 0.47) between intentions and behavior.

However, direct evidence of whether situational interviews predict later performance because they actually measure behavioral intentions is absent from the literature. Thus far, the only indirect evidence for the behavioral intentions explanation comes from Sue-Chan et al. (1995), who found a positive correlation between self-efficacy and situational interview performance. Self-efficacy refers to beliefs about one’s own capability to perform certain behavior even in the face of obstacles or barriers (Bandura 1986). Yet, a positive correlation between self-efficacy and situational interview performance does not provide convincing evidence that situational interviews measure behavioral intentions. If the behavioral intentions explanation is correct, self-efficacy should also predict future performance directly and moderate the relationship between the intentions stated during the situational interview and future performance (e.g., Armitage and Conner 2001; Terry and O’Leary 1995).

A direct test of the suggestion that situational interviews are criterion valid because they assess behavioral intentions require that interviewees take part in a situational interview and are subsequently faced with situations that are in fact similar to the situations described in the interview. This would make it possible to observe their actual behavior in corresponding work-related situations and to test whether this behavior is similar to what they said they would do in the interview. Accordingly, our first aim was to examine the actual similarity between what interviewees say they would do in the situations presented to them during a situational interview (i.e., their intentions) and their actual behavior when they are confronted with corresponding work-related situations. Based on Latham et al.’s (1980) arguments, we hypothesize the following:

### **Hypothesis 1a**

What interviewees say they would do in situational interviews is similar to their actual behavior in corresponding work-related situations.

As the hypothetical situations presented during the situational interview are most likely new to the interviewees, situational interviews not only force interviewees to state their intentions, they also force interviewees to *form* specific intentions as to what they would do in particular situations. The formation of intentions may create a sense of commitment to the behavior and also an association between specific aspects of the situation (e.g., a specific complaint of a client) and the behavior (Webb and Sheeran 2008). Therefore, if situational interviews do capture intentions, their predictiveness should be especially high for situations that are similar to the situations described during the interview (i.e., corresponding work-related situations) and low for situations they have not been confronted with during the interview (i.e., non-corresponding work-related situations). Therefore, we hypothesize the following:

### **Hypothesis 1b**

The correlation between performance in the situational interview and performance in a job simulation is higher for corresponding work-related situations compared to non-corresponding work-related situations.

According to the theory of planned behavior (Ajzen 1991), perceived behavioral control is considered to affect intentions, have a direct effect on behavior, and moderate the intentions-behavior relationship. In the present study, we focus on the direct effect of perceived behavioral control on behavior and its moderation effect on the intentions-behavior relationship, as these two effects are considered to be particularly relevant in the prediction of behavior under low volitional control as is the case with work-related performance (Armitage and Conner 2001). In line with previous research, perceived behavioral control is operationalized by two variables: confidence in the capability to perform the behavior (i.e., self-efficacy) and the belief that the outcome can be influenced by one’s own efforts (i.e., perceived control). This distinction should be made since we cannot assume that an individual’s perception of the extent to which a behavior would be influenced by one’s own efforts corresponds with their judgments as to how easy that behavior would be to perform (Terry and O’Leary 1995). Intentions and perceived behavioral control are expected to interact in predicting performance based on the following rationale: no matter how strong intentions are, the implementation of an intention into action is at least partially determined by personal and environmental barriers. Thus, in line with the theory of planned behavior, we hypothesize the following:

### **Hypothesis 2a**

Perceived behavioral control moderates the relationship between performance in the situational interview and performance in corresponding work-related situations, so that the relationship is stronger when interviewees’ perceived behavioral control is high than when interviewees’ perceived behavioral control is low.

Furthermore, perceived behavioral control is held to exert a direct effect on behavior. Thus, if the behavioral intentions explanation for situational interview validity is correct, perceived behavioral control not only moderates the intentions-behavior relationship, but it should also predict behavior directly. Accordingly, we suggest

### **Hypothesis 2b**

Perceived behavioral control is positively related to interviewees’ performance in a job simulation.

## Interviewees’ Ability to Identify Criteria and the Validity of Situational Interviews

Recently, Kleinmann et al. (2011) presented another explanation for the criterion-related validity of personnel selection procedures in general, including situational interviews. Their explanation assumes that individuals actively strive to successfully handle the situations that they are faced with during the selection procedures, so as to attain positive evaluations. According to Kleinmann et al., this ATIC refers to whether individuals are able to correctly decipher the situational demand characteristics and use them to guide their behavior. ATIC reflects an ability that not only helps individuals to better read the situational demands in interviews, but also those in work contexts. Thus, situational interviews predict performance because they capture whether interviewees are able to read situational demands—or in other words know how they should behave to master performance-relevant situations—both during the interview and on the job (cf. Ingold et al. 2015; Jansen et al. 2013). Thus, for this explanation, it is relevant that ATIC as a common cause is positively related to both performance in the interview and performance in work-related situations. Thereby, ATIC contributes to the criterion-related validity of situational interviews because these interviews capture interviewees standing on this general ability that helps individuals to better read the situational demands in varying social situations, including selection and job contexts.

It has already been shown that the correct perception of situational demands correlates with performance in personality questionnaires, assessment centers, and also situational interviews (e.g., Griffin 2014; Ingold et al. 2015; Jansen et al. 2012; König et al. 2007; Melchers et al. 2009). In line with previous findings, we therefore suggest the following hypothesis:

### **Hypothesis 3**

There is a positive relationship between ATIC, as measured in the situational interview, and performance in the situational interview.

According to Kleinmann et al. (2011), ATIC scores from situational interviews should predict performance in other work-related situations. König et al. (2007) already found that ATIC scores from a structured interview were predictive of performance in an assessment center and vice versa (*r* = 0.29 and *r* = 0.34, respectively). Furthermore, recently Jansen et al. (2013) found that ATIC scores derived from an assessment center predicted actual job performance (*r* = 0.27), and Ingold et al. (2015) found that ATIC scores derived from a situational interview predicted supervisor ratings of job performance (*r* = 0.29). Therefore, we hypothesize the following:

### **Hypothesis 4**

There is a positive relationship between ATIC as measured in the situational interview and performance in work-related situations.

If the explanation by Kleinmann et al. (2011) for the criterion-related validity of situational interviews is correct, then ATIC should contribute to their criterion-related validity. In other words, individual differences in the ability to read situational demands should at least partly explain why situational interviews predict performance in work-related situations. In line with this, the following is hypothesized:

### **Hypothesis 5**

The relationship between performance in the situational interview and performance in a job simulation decreases when ATIC is taken into account.

## Method

### Sample

We recruited 101 students (70 females and 31 males) enrolled in various graduate and undergraduate courses at a large Dutch university, who participated in a selection simulation. Their mean age was 22.33 years (*SD* = 2.31) and their job experience varied between 6 months and 16 years (*M* = 4.86 years, *SD* = 3.23). Power analyses (Faul et al. 2009) showed that a minimum sample size of 84 was needed to detect medium-sized direct effects (*r* = 0.30), a minimum sample size of 99 was needed to detect small differences in correlation coefficients (Δ*r* = 0.20) for highly correlated coefficients (*r* = 0.70), and a minimal sample size of 92 was needed to detect small increases in explained variance in the regression models (*f*^2^ = 0.15), with an *α* of 0.05.

### Procedure

The selection simulation mirrored the selection procedure of a sales manager. This position was chosen because it represents the most popular student job in the Netherlands (Central Bureau of Statistics 2013). To make the simulation more intrinsically motivating for participants, only students with sales experience were allowed to participate. Prior to the selection simulation, participants received a hypothetical job advertisement for a sales manager position (see Appendix) and were asked to prepare accordingly. To further motivate participants, they were informed that a professional report of their test scores would be sent to them after the selection simulation and that a cash prize of €50 (equal to $64) would be given to the best interviewee.

**Fig. 1:**
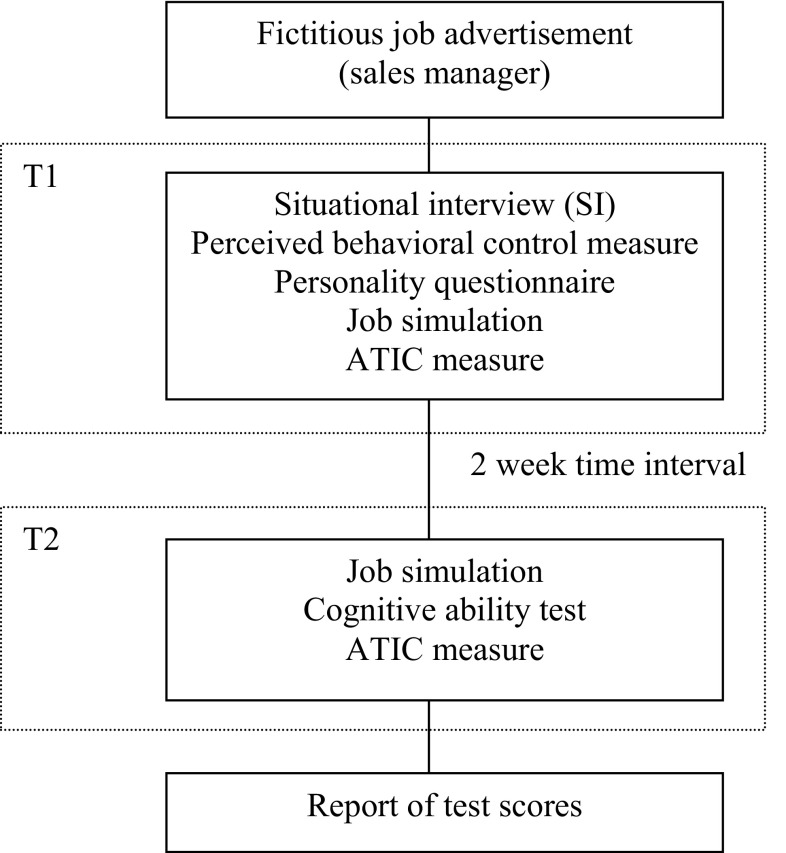
Procedure and timeline

The selection simulation lasted 8 h spread over 2 days that were 2 weeks apart (cf. Fig. 1). On the 1st day, interviewees took part in a situational interview and were then faced with a job simulation containing corresponding as well as non-corresponding work-related situations to be able to observe their actual behavior (see below for more information concerning the development of the interview and the job simulation). The reason why both types of situations were included was twofold. First, it helped make the goal of the study less obvious to participants. Consequently, it helped prevent participants to actively try to remember their earlier responses during the interview and behave accordingly. Second, to test whether situational interviews make interviewees form behavioral intentions as to what they would do in particular situations, we needed to compare the predictiveness of the situational interview for behavior in both corresponding and non-corresponding situations.

We used a job simulation to observe participants’ actual behavior not only because it allowed us to include corresponding and non-corresponding situations, but also because simulations are based on the notion of behavioral consistency so that interviewees’ performance in the simulation is assumed to be consistent with their on-the-job behavior (Motowidlo et al. 1990; Wernimont and Campbell 1968). To this end, simulations aim to maximize the point-to-point correspondence with the criterion (Lievens and De Soete 2012). This particular simulation has many characteristics of a typical performance measure, because it correlates with personality, but not with cognitive ability (e.g., De Soete et al. 2013; Oostrom et al. 2011). Furthermore, scores on this type of job simulation have been found to predict several work-related variables (Lievens et al. in press; Oostrom et al. 2010, 2011).

To provide a more conservative test of the relationship between performance in the situational interview and performance in the job simulation, a second job simulation was administered 2 weeks later, which allowed us to check whether participants’ responses to the situational interview questions and their actual behavior were similar simply because they remembered the answers they had just provided during the interview. If situational interview validity would be a memory phenomenon, the answers during the situational interview and the behaviors shown during the simulation at T2 should hardly show any similarity.

**Fig. 2:**
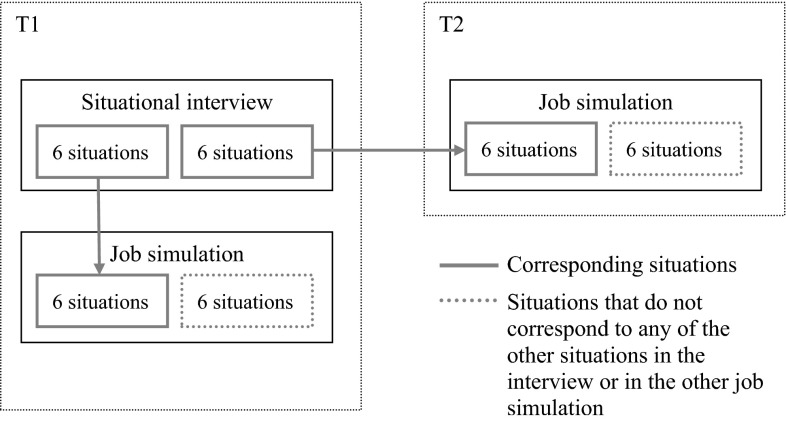
Overview of corresponding and non-corresponding situations in the situational interview and the job simulations. The *arrows* show which situations in the interview corresponded to which situations in the job simulations

Specifically, during the situational interview, each interviewee was presented with 12 situations, half of which were again presented in the job simulation at T1 and the other half in the job simulation at T2. Both simulations contained six different, additional situations that did not correspond with the situations that were presented during the interview (see Fig. 2). The same set of interview questions as well as work-related situations in the job simulations was used for all interviewees.

During the situational interview, interpersonal situations were described and participants had to state how they would react if they actually found themselves in these situations. Furthermore, in the job simulations, the situations were presented via video clips. In these video clips, an actor looked directly into the camera and addressed the participants, who then had to respond as if they were actually talking to the actor. The participant had to respond as if it was a real situation. These responses were recorded with a webcam. The job simulations were designed to mimic psychological and physical key aspects of the job of a sales manager.

At the end of each day, participants received the ATIC questionnaire that presented them with the situations in the interview and the job simulation in which they had participated in before (18 in total, since 6 of the 12 situations in the interview corresponded with 6 of the 12 situations in the job simulation, these 6 were presented only once in the ATIC questionnaire). For each situation, they had to write down their assumptions about the targeted dimensions. Participants were asked to give behavioral examples for their assumptions. They were encouraged to write as many dimensions (e.g., creativity) and behavioral examples (e.g., coming up with new ideas, thinking outside the box) per situation as they could think of. To ensure that participants understood this procedure, they received an example. Furthermore, at T1, participants also completed an online perceived behavioral control measure.

### Development of the Situational Interview and the Job Simulations

The situations in the situational interview and job simulations were developed by a management consultancy. This was done in line with existing procedures for constructing simulations, which start with a job analysis (Chan and Schmitt 1997; Weekley and Jones 1997). Critical incident interviews were conducted with 15 experienced sales people and managers at different companies (e.g., an engineering agency, a job consultancy, a government institution, and a retailer). Based on these interviews, scenarios of work-related interpersonal situations were written. The relevance and suitability of each item was evaluated by the same experienced sales people and managers. The scenarios that survived this step were subsequently videotaped by a professional film company. The items were clustered based on their content and pilot data, which resulted in six dimensions aimed to measure self-control, client orientation, persuasiveness, perseverance, initiating structure, and consideration.

In line with previous studies (e.g., Griffin 2014; Jansen et al. 2013; Melchers et al. 2009), the situations were then pretested to examine whether they did indeed reflect the targeted dimensions. Four subject matter experts (one female and three males; age *M* = 41.00, *SD* = 13.33), with an average job experience of 19.75 years (*SD* = 9.84) in human resource management and/or test development, each rated the degree to which the situations reflected the six dimensions on a five-point scale (1 = *not at all*, 5 = *fully*). Sufficient agreement was found between the experts, as indicated by a one-way random effects intraclass correlation (ICC) for consistency of 0.77 (cf. McGraw and Wong 1996). Only situations rated as clearly measuring the intended dimensions (*M* = 4.50 or higher) and none of the non-intended dimensions were chosen. For these situations, the mean correlation coefficient between the four experts was 0.54 and the corresponding ICC was 0.88. We also asked the experts to indicate for each situation whether it would be measuring a job dimension other than the six intended job dimensions. The experts did not indicate an alternative job dimension for any of the situations.

### The Rating Process

Four student assistants (two students who were in their 3rd year of a full-time psychology program and two graduate students; three females and one male), who were enrolled in advanced Work and Organizational Psychology courses, received a 4-h frame-of-reference training (Roch et al. 2012). In the training, they were introduced to basics of rating processes, the situational interview, the job simulation, and the ATIC measure as well as to definitions and examples of poor, moderate, and high-scoring interviewees on the dimensions to be assessed. They practiced the rating process, worked with the scoring instructions, discussed their ratings, and received feedback on their ratings. Furthermore, the assistants were introduced to the other measures included in the selection simulation.

All ratings were provided by two randomly selected student assistants out of the pool of four. For each part of the selection simulation (i.e., conducting and rating the interview, rating the job simulation, rating the ATIC measure, and the administration of the questionnaires), a different pair of student assistants was selected. The two raters gave their ratings independently of one another. All ratings (except for the ATIC and the similarity ratings—see below) were on a five-point scale ranging from 1 = *very ineffective* to 5 = *very effective*. When their ratings differed by more than one point, they discussed their observations and adjusted their ratings accordingly.

During the interview, one of the student assistants presented the questions and the other one recorded participants’ answers to be able to later score the similarity with the responses in the job simulations. The student assistants were instructed to read the interview questions as printed on the forms and not to rephrase them or give additional cues. The research assistants were blind to the purpose of the study and the purpose of the similarity ratings.

## Measures

### Situational Interview

Following Chan and Schmitt (1997) and Lievens and Sackett (2006), the situations in the job simulation were used to develop the situational interview. An example of a situational interview question is: “One of your employees is misbehaving: He shirks his assigned duties, and when he does carry out his duties he makes a lot of mistakes and doesn’t finish them. You have already discussed this problem with the employee several times. He has reached your limit and you have asked him to come to your office. The employee asks you what you want to talk to him about. What would you do?” In line with previous studies (e.g., Conway et al. 1995), the coefficient alpha for the interview was high (*α* = 0.88). To determine interrater reliability, we calculated a one-way random effects ICC for consistency for the interview rating across the 12 interview questions. This ICC was 0.85 and the mean correlation between the raters was 0.71. The individual raters’ means varied between 2.92 (*SD* = 1.00) and 3.08 (*SD* = 0.98).

### Similarity Rating

After the data were collected, the student assistants used the notes taken during the situational interviews and individually rated the similarity between the interview answers and participants’ actual behavior during the job simulation on a five-point scale ranging from 1 = *very different* to 5 = *highly similar*. The one-way random effects ICC coefficient for consistency was 0.92 and the mean correlation coefficient between the raters was 0.81 at T1 and 0.80 at T2, which again represents good interrater reliability. The individual rater’s means varied between 3.11 (*SD* = 1.19) and 3.37 (*SD* = 1.21) at T1 and between 3.24 (*SD* = 1.13) and 3.43 (*SD* = 1.12) at T2.

### Perceived Behavioral Control

In line with Manstead and Van Eekelen (1998), perceived behavioral control was operationalized as confidence in the ability to perform the behavior (self-efficacy) and the belief that the outcome can be influenced by one’s own efforts (control). Self-efficacy was measured with the following three items adopted from Manstead and Van Eekelen: “I am certain that I can perform well in similar situations” (1 = *completely disagree*, 7 = *completely agree*), “How confident are you that you will perform well in similar situations?” (1 = *very little,* 7 = *a great deal*), “To perform well in similar situations is… for me” (1 = *very difficult*, 7 = *very easy*). Control was measured with the following three items adopted from Manstead and Van Eekelen: “Whether or not I perform well in similar situations is completely up to me” (1 = *completely disagree*, 7 = *completely agree*), “How much control do you have over whether you perform well in similar situations?” (1 = *none*, 7 = *complete*), “There is a lot that I can do to be sure of that I perform well in similar situations” (1 = *completely disagree*, 7 = *completely agree*). Coefficient alpha was 0.83 for self-efficacy and 0.78 for control. The correlation between the two scales was 0.41 (*p* < 0.01).

### Job Simulation

An example of a situation in the job simulation corresponding to the situational interview question is: “Narrative: One of your employees is misbehaving: He shirks his assigned duties, and when he does carry out his duties he makes a lot of mistakes and doesn’t finish them. You have discussed this problem with the employee several times already. He has reached your limit and you have asked him to come to your office. Employee: You wanted to talk to me about something. What’s it about?” Coefficient alpha was 0.84 at T1 and 0.83 at T2. The one-way random effects ICC for consistency for the mean ratings across the 12 situations was 0.91 at T1 and 0.90 at T2.

### ATIC

In line with previous research (e.g., Ingold et al. 2015; Jansen et al. 2013), ATIC was measured by the degree to which each of the participants’ assumptions and behavioral examples corresponded to the targeted dimensions. ATIC was evaluated on a four-point scale ranging from 0 = *no fit* to 3 = *fits completely*. To be able to test our hypotheses, we calculated two ATIC scores: one score based on the participants’ assumptions of what the 12 situational interview questions were measuring and one score based on participants’ assumption of what the 24 situations in the two job simulations were measuring. Coefficient alpha was 0.72 for the ATIC measure from the situational interview and 0.83 for the ATIC measure from the job simulation. The correlations between the different ATIC measures provide the opportunity to calculate alternate forms reliability coefficients for the non-corresponding situations, which varied between 0.56 and 0.67 (corrected for test length), and test–retest reliability (with a time lag of 2 weeks) for the corresponding situations at T1 and T2, which was 0.63 (corrected for test length). The one-way random effects ICC for consistency was 0.91 for the ATIC measure from the situational interview and 0.92 for the ATIC measure from the job simulations. The mean correlation coefficient between the raters was 0.82 for ATIC based on the situational interview and 0.70 for ATIC based on the job simulations. The individual rater’s means varied between 0.57 (*SD* = 0.85) and 0.79 (*SD* = 1.01) for ATIC based on the situational interview and between 0.55 (*SD* = 0.82) and 0.64 (*SD* = 0.95) for ATIC based on the job simulations.

### Other Variables

To be able to check the external validity of the selection simulation, motivation and perceived realism were measured at T1 and T2. Participants rated these items on a scale ranging from 1 = *strongly disagree* to 5 = *strongly agree*. Motivation was measured with five items adopted from Arvey et al. (1990). An example of an item is: “I wanted to do well on the selection simulation.” Coefficient alpha was 0.76 at T1 and 0.75 at T2. Perceived realism was measured with the following two items: “Did you act like a real applicant in the selection simulation” and “Did you perceive the selection simulation to be realistic.” Coefficient alpha for this two-item scale was 0.63 at T1 and 0.70 at T2.

## Results

We first looked at participants’ scores on motivation and perceived realism. The mean scores on motivation (T1: *M* = 3.86, *SD* = 0.60 and T2: *M* = 3.81, *SD* = 0.61) and perceived realism (T1: *M* = 3.44, *SD* = 0.74, 54.4 % agreed or strongly agreed that the situations were realistic and another 26.7 % showed moderate agreement, and T2: *M* = 3.46, *SD* = 0.72, 56.4 % agreed or strongly agreed that the situations were realistic and another 27.7 % showed moderate agreement) showed that participants were motivated to perform well and perceived the selection situation as relatively realistic. Participants’ motivation and perceived realism did not differ significantly between T1 and T2 (both *t*s < 1).

Table 1 shows means, *SD*s, reliabilities (coefficient alphas), and correlations between all study variables. Situational interview performance was significantly correlated with performance in the job simulation at T1 (*r* = 0.67, *p* < 0.01) and the job simulation at T2 (*r* = 0.58, *p* < 0.01).

**Table 1:** Means, SDs, reliabilities, and correlations of all study variables

	*M*	*SD*	1	2	3	4	5	6	7	8	9
Self-efficacy	4.90	0.78	(0.83)								
Control	5.21	0.86	0.41**	(0.78)							
Situational interview	2.98	0.50	0.31**	0.07	(0.88)						
Job simulation (T1)	2.92	0.50	0.14	0.09	0.67**	(0.84)					
Job simulation (T2)	3.09	0.44	0.28**	0.06	0.58**	0.72**	(0.83)				
Job simulation (corresponding)	3.03	0.46	0.25*	0.16	0.64**	0.91**	0.85**	(0.82)			
Job simulation (non-corresponding)	2.99	0.47	0.18	0.00	0.65**	0.88**	0.88**	0.80**	(0.82)		
ATIC interview	0.58	0.33	−0.12	−0.13	0.33**	0.48**	0.39**	0.43**	0.47**	(0.72)	
ATIC simulation	0.48	0.30	−0.12	−0.10	0.34**	0.46**	0.40**	0.43**	0.45**	0.82**	(0.83)

*N* = 101. Coefficient alphas are reported on the diagonal within parentheses. Self-efficacy and control were measured on a seven-point scale. Performance on the situational interview and on the job simulations were measured on a five-point scale and ATIC (= ability to identify criteria) was scored on a four-point scale. Job simulation (corresponding) represents the combined score of the six situations in the job simulation at T1 and the six simulations at T2 that corresponded with the 12 situations in the interview. Job simulation (non-corresponding) represents the score on the six situations in the job simulation at T1 and the six situations at T2 that did not correspond with the situations in the interview

* *p* < 0.05, ** *p* < 0.01

## Tests of Hypotheses Concerning the Behavioral Intention Explanation

Hypothesis 1a posited that what interviewees say they would do in situational interviews is similar to their actual behavior in corresponding situations. To test this hypothesis, we looked at how similar the answers to the situational interview questions were to the behavioral responses in the corresponding situations in the two job simulations. We found a mean similarity rating of 4.00 (*SD* = 0.44) for the six corresponding situations in the job simulation at T1 and a mean similarity rating of 3.46 (*SD* = 0.64) for the six corresponding situations in the job simulation at T2. These similarity ratings were both much closer to the high end point of the scale (5) than to the low end point (1) and were also significantly higher than the mid-point of the scale, *t*(96) = 17.82 and 7.16 for T1 and T2, both *ps* < 0.01. Based on these findings, Hypothesis 1a was supported.

It turned out that the similarity ratings at T1 were significantly higher than at T2, *t*(96) = 5.62, *p* < 0.01, *d* = 0.98, suggesting that participants’ memory of their answers from the interview seems to influence their behavior in the simulation. Nevertheless, the substantial similarity between their answers from the situational interview and their behavior 2 weeks later supports the argument that what people do is similar to what they say they would do even when they are less able to recall their exact answers.

Hypothesis 1b stated that the correlation between performance in the situational interview and performance in the job simulation would be higher for corresponding than for non-corresponding work-related situations. We tested whether the correlation between scores on the six corresponding situations in the situational interview and the job simulation at T1 was higher than the correlation between scores on these same six situational interview questions and the non-corresponding situations at T1. However, in contrast to our hypothesis, the correlations for corresponding and non-corresponding situations (*r*s = 0.64 and 0.58, both *p*s < 0.01, respectively) did not differ significantly, *z* = 1.00, *p* = 0.16. A similar pattern was found for the correlations between the other six situational interview questions and the corresponding situations and non-corresponding situations in the job simulation at T2, both *r*s = 0.49, *p*s < 0.01, *z* = 0.00, *p* = 0.50. Thus, Hypothesis 1b was not supported.

Hypothesis 2a stated that perceived behavioral control would moderate the relationship between performance in the situational interview and performance in corresponding situations. To test this hypothesis, we conducted two hierarchical regression analyses with situational interview performance, self-efficacy, and control in Step 1 and the products of situational interview performance and self-efficacy and situational interview performance and control in Step 2 (cf. Table 2). As the situational interview had the same predictiveness for behavior in corresponding and non-corresponding job situations and the pattern of correlations was the same for the job simulation at T1 and at T2, the hypothesis was tested for the 12 corresponding and the 12 non-corresponding situations in the job simulations. No significant interaction effects were found which means that the hypothesis was not supported.

**Table 2:** Standardized regression weights and explained variances for the moderation effect of perceived behavioral control on the relationship between performance in the situational interview and the job simulations

	Corresponding situations in job simulations		Non-corresponding situations in job simulations	
	Step 1	Step 2	Step 1	Step 2
Situational interview performance (SI)	0.63**	0.64**	0.66**	0.68**
Self-efficacy	−0.01	0.03	−0.03	−0.01
Control	0.10	0.07	−0.05	−0.05
SI × self-efficacy		0.11		0.03
SI × control		−0.14		−0.09
Total *R* ^2^	0.41**	0.42**	0.43**	0.43**
Δ*R* ^2^		0.01		0.01

*N* = 101. Δ*R* ^2^ may appear inconsistent due to rounding

** *p* < 0.01

Hypothesis 2b, which stated that perceived behavioral control, operationalized as self-efficacy and control, would be positively related to interviewees’ performance in a job simulation, was partially supported. Self-efficacy significantly correlated with performance in the 12 situations in the job simulations that corresponded with the interview situations (*r* = 0.25, *p* < 0.05), but control did not (*r* = 0.16, *p* = 0.13). No significant correlations were found between self-efficacy and control on the one hand and performance in the 12 non-corresponding situations in the job simulations on the other hand (*r* = 0.18 and 0.00, both *p*s > 0.07, respectively).

## Tests of Hypotheses Concerning the ATIC Explanation

In line with Hypothesis 3, which predicted that there would be a positive relationship between ATIC as measured in the situational interview and performance in the situational interview, we found a significant correlation of *r* = 0.33, *p* < 0.01, between ATIC in the interview and interview performance.

Hypothesis 4, which stated that there would be a positive relationship between ATIC as measured in the situational interview and performance in a job simulation, was also supported. At T1, ATIC from the situational interview correlated *r* = 0.48 (*p* < 0.01) with performance in the job simulation. At T2, ATIC from the situational interview correlated *r* = 0.39 (*p* < 0.01) with performance in the job simulation.

Hypothesis 5 predicted that the relationship between performance in the situational interview and performance in the job simulation decreases when ATIC is taken into account. To test this hypothesis, we calculated the partial correlation between participants’ performance in the situational interview and their performance in corresponding situations in the job simulation by partialling out both ATIC scores. The partial correlation was *r* = 0.56, *p* < 0.01. To test whether the partial correlation was significantly lower than the zero-order correlation of *r* = 0.64, *p* < 0.01, we used a procedure suggested by Olkin and Finn (1995) and later extended by Graf and Alf (1999). This procedure revealed that the 95 % confidence interval (CI) for this difference did not include zero but ranged from 0.005 to 0.128. Similarly, the partial correlation of performance in the interview and performance in non-corresponding situations in the job simulation (*r* = 0.58, *p* < 0.01) was significantly lower than the zero-order correlation (*r* = 0.65, *p* < 0.01, CI for the difference = 0.002–0.122). Thus, in line with Hypothesis 5, statistically controlling for ATIC from the situational interview significantly lowered the validity of the situational interview.

Following Jansen et al. (2013), we conducted another test of Hypothesis 5 and used structural equation modeling to test whether ATIC is a common cause of both performance in the interview and performance on the job simulations. For the model test, ATIC, situational interview performance, and performance in the job simulations were each defined by two parcels of items, one for the corresponding and one for the non-corresponding items. The model with a direct path from ATIC to both situational interview performance and performance in the job simulations had a very good fit, *χ*^2^(6) = 27.77, *p* < 0.01, CFI = 0.99, TLI = 0.97, RMSEA = 0.07, SRMR = 0.02. The path from ATIC to situational interview performance was 0.43 (*p* < 0.01), the path from ATIC to performance in the job simulations was 0.38 (*p* < 0.01), and the path from situational interview performance to performance in the job simulations was 0.59 (*p* < 0.01). We tested an additional model that did not include a direct path from ATIC to performance in the job simulation. In this model, the path from ATIC to situational interview performance was 0.48 (*p* < 0.01), which was rather similar to the previous model, but the path from situational interview performance to performance in the job simulations was 0.77 (*p* < 0.01), which was much larger than in the previous model. This model had a worse fit, Δ*χ*^2^(1) = 83.15, *p* < 0.01, *χ*^2^(7) = 110.92, *p* < 0.01, CFI = 0.95, TLI = 0.90, RMSEA = 0.15, SRMR = 0.08. These results show that that the common cause model is more appropriate and that the path between situational interview performance and performance in the job simulations becomes much weaker when ATIC is taken into account as a common cause.

## Discussion

Although situational interviews are a valid predictor of job performance, the underlying reasons for why they predict performance have not been resolved, yet. The present study made an effort to open the ‘black box’ of interview validity by examining two explanations for their validity, namely (a) that the situational interview measures interviewees’ behavioral intentions (e.g., Latham 1989; Latham et al. 1980) and (b) that situational interviews measures whether interviewees are able to correctly decipher the situational demands they are faced with in social situations (cf. Kleinmann et al. 2011).

We provided the first direct test of the behavioral intentions explanation of situational interview validity. In support of this explanation, we found considerable similarity in what interviewees say they would do and their actual behavior in corresponding situations. Furthermore, we replicated Sue-Chan et al.’s (1995) finding of a positive relationship between self-efficacy and interview performance. In addition, we found that self-efficacy was also positively related to performance on the job simulation. Yet, this last finding would also have been predicted by the second explanation.

In contrast to the behavioral intentions explanation, our results indicated that perceived control did not affect situational interview performance and that neither self-efficacy nor control moderated the relationship between situational interview performance and performance on the job simulation. Although we found that the content of interviewees’ answers to the situational interview questions was similar to their behaviors when confronted with the same situations in a job simulation, the validity for the situational interview was just as high when the situations in the interview and in the job simulation did not correspond. If situational interviews do capture intentions, their validity should have been higher for corresponding situations compared to non-corresponding situations. Hence, we believe our findings stress that situational interviews are measuring some valuable performance-related information beyond or in addition to behavioral intentions.

Our results supported the role of ATIC for situational interview validity: ATIC was a significant predictor of performance in situational interviews and job simulations. Furthermore, ATIC explained part of the validity of the situational interview, so that the correlation between situational interview performance and performance in the simulations dropped when ATIC was partialled out from this relationship. These findings add to the evidence that the assessment of situational demands explains part of the validity of these selection instruments (e.g., Ingold et al. 2015; Jansen et al. 2013). For the ATIC explanation for situational interview validity, it did not matter whether interviewees’ actual behaviors were in line with the intentions they conveyed during the interview, because ATIC reflects a general ability that helps individuals to better read the situational demands in varying social situations, including selection and job contexts. Our results supported this view of ATIC as a more general ability, as ATIC from the interview predicted behavior equally well in corresponding as well as non-corresponding situations in the job simulation.

Concerning the practical implications of these findings, organizations might consider using ATIC as part of the selection procedure as our results showed, in line with previous studies (Ingold et al. 2015; Jansen et al. 2013), that situation perception is related to behavior in work-related situations. Such a test could easily be administered by asking interviewees what they thought was assessed in the situational interview and/or in other assessment instruments used for selection decisions (e.g., Kleinmann et al. 2011; Jansen et al. 2013). Assessing interviewees’ ATIC might be of additional use for making these decisions, particularly for jobs where it is essential to assess situational demands.

The present study has some limitations that should be noted. First, our data were obtained from a sample of students and the selection procedure was simulated. We chose such a selection simulation because the test of the behavioral intention explanation required interviewees to take part in a situational interview and then be faced with both corresponding and non-corresponding situations in which their actual behavior could be observed. Furthermore, the selection simulation allowed us to assess all relevant variables in a standardized way. Furthermore, despite the relatively low incentive (i.e., a professional report of their test scores and a cash prize of $64 for the best interviewee), most participants perceived the selection simulations as relatively realistic and they were motivated to perform well.

A second limitation is that we used a high-fidelity job simulation instead of actual job performance data. A direct test of the idea that situational interviews are criterion valid because they assess behavioral intentions would require that participants take part in a situational interview and are subsequently faced with similar situations on the job. Unfortunately, it would be practically impossible to present participants with the exact same situations on their actual job. Furthermore, simulations are based on the notion of behavioral consistency: performance in the simulation is assumed to be consistent with on-the-job behavior (Motowidlo et al. 1990; Wernimont and Campbell 1968). Simulations have traditionally been categorized as scoring high on fidelity, as they present work-related situations and require actual behavioral responses (Thornton and Rupp 2006). Furthermore, the simulations used in the present study have been found to predict several work-related variables (Lievens and De Soete 2012; Oostrom et al. 2010, 2011). For these reasons, we believe that participants’ behavior during the job simulation reflects how they would behave on the job.

Third, we cannot rule out memory effects despite the time interval of 2 weeks. Although the similarity ratings between the answers during the situational interview and the job simulation at T2 were lower than the similarity between the answers during the situational interview and the job simulation at T1, our time interval might have been too short for participants to completely forget the answers they gave during the situational interview at T1. Thus, the validity of the situational interview could partly be due to participants’ recall of their answers. However, the correlations between the scores on the situational interview and behavior in corresponding and non-corresponding situations in the job simulation at T1 were not significantly different. Thus, the predictiveness of the situational interview was as high for corresponding situations as for situations in the job simulation that were not presented before. A similar pattern was found for the correlations between scores on the situational interview and the job simulation at T2. Furthermore, we did not instruct participants to act in line with their answers to the interview. When asked whether they had an idea about the goal of the study, only six participants (5.66 %) mentioned the corresponding situations in the interview and the job simulation. Therefore, we believe it is unlikely that memory played a large role in our findings. Nevertheless, we suggest further research using a larger time interval.

A final limitation concerns the limited power in the present study to test the moderation effect related to the behavioral intentions explanation. Even though we had sufficient power to test the different main effects, interaction effects suffer from much lower power for samples sizes like those used for the present research (Aguinis 2002). However, even when we only consider the results for the main effects, the present study found more evidence for the ATIC explanation than for the behavioral intention explanation for situational interview validity. Nevertheless, we urge further studies on the behavioral intention explanation. Sheppard et al.'s (1988) meta-analysis showed that measures of self-predictions have stronger relationships with behavior than with behavioral intentions. Therefore, we advocate examining these self-predictions in future research. Another avenue for future research could be to measure the stability of the intentions (e.g., by asking the situational interview questions twice). Several studies showed that the impact of intentions on behavior is moderated by intention stability such that intentions with greater stability are more predictive of future behavior (e.g., Conner et al. 2000).

## Appendix: Hypothetical Job Advertisement for a Sales Manager Position

### Retail Sales Manager

As a sales manager you are responsible for the success of the store. You build, inspire, and supervise a team of 5–15 employees to deliver measurable results. By training and coaching your employees, you will encourage their growth. You are capable of translating the store’s vision to the daily practice. You ensure that the back-office and appearance of the store remains up-to-date. As a sales manager you are expected to be present in the store and help your team deliver positive experiences for customers, as they shop and get support. You will report back to the regional manager. Sales is one of your core competencies and the store targets and aligned bonuses motivate you to take the success of your team to the next level.

You are the manager with a vision and a proactive attitude that sees and thinks in opportunities and solutions.
